# CCL2 modulates cytokine production in cultured mouse astrocytes

**DOI:** 10.1186/1742-2094-7-67

**Published:** 2010-10-14

**Authors:** Bridgette D Semple, Tony Frugier, M Cristina Morganti-Kossmann

**Affiliations:** 1 National Trauma Research Institute, The Alfred Hospital and Department of Medicine, Monash University, Melbourne, Victoria, Australia; 2 Anatomical Pathology Department, The Alfred Hospital, Melbourne, Victoria, Australia

## Abstract

**Background:**

The chemokine CCL2 (also known as monocyte chemoattractant protein-1, or MCP-1) is upregulated in patients and rodent models of traumatic brain injury (TBI), contributing to post-traumatic neuroinflammation and degeneration by directing the infiltration of blood-derived macrophages into the injured brain. Our laboratory has previously reported that *Ccl2*-/- mice show reduced macrophage accumulation and tissue damage, corresponding to improved motor recovery, following experimental TBI. Surprisingly, *Ccl2*-deficient mice also exhibited delayed but exacerbated secretion of key proinflammatory cytokines in the injured cortex. Thus we sought to further characterise CCL2's potential ability to modulate immunoactivation of astrocytes *in vitro*.

**Methods:**

Primary astrocytes were isolated from neonatal wild-type and *Ccl2*-deficient mice. Established astrocyte cultures were stimulated with various concentrations of lipopolysaccharide (LPS) and interleukin (IL)-1β for up to 24 hours. Separate experiments involved pre-incubation with mouse recombinant (r)CCL2 prior to IL-1β stimulation in wild-type cells. Following stimulation, cytokine secretion was measured in culture supernatant by immunoassays, whilst cytokine gene expression was quantified by real-time reverse transcriptase polymerase chain reaction.

**Results:**

LPS (0.1-100 μg/ml; 8 h) induced the significantly greater secretion of five key cytokines and chemokines in *Ccl2*-/- astrocytes compared to wild-type cells. Consistently, IL-6 mRNA levels were 2-fold higher in *Ccl2*-deficient cells. IL-1β (10 and 50 ng/ml; 2-24 h) also resulted in exacerbated IL-6 production from *Ccl2*-/- cultures. Despite this, treatment of wild-type cultures with rCCL2 alone (50-500 ng/ml) did not induce cytokine/chemokine production by astrocytes. However, pre-incubation of wild-type astrocytes with rCCL2 (250 ng/ml, 12 h) prior to stimulation with IL-1β (10 ng/ml, 8 h) significantly reduced IL-6 protein and gene expression.

**Conclusions:**

Our data indicate that astrocytes are likely responsible for the exacerbated cytokine response seen *in vivo *post-injury in the absence of CCL2. Furthermore, evidence that CCL2 inhibits cytokine production by astrocytes following IL-1β stimulation, suggests a novel, immunomodulatory role for this chemokine in acute neuroinflammation. Further investigation is required to determine the physiological relevance of this phenomenon, which may have implications for therapeutics targeting CCL2-mediated leukocyte infiltration following TBI.

## Background

Cerebral inflammation involving the release of soluble mediators, infiltration of peripheral immune cells and activation of resident glial cells, is one of the key pathophysiological processes contributing to secondary degeneration following focal traumatic brain injury (TBI). The chemokine CCL2 (also known as macrophage chemoattractant protein-1, or MCP-1) is well recognised for its potent ability to mediate macrophage recruitment and migration to sites of inflammation [1,2]. In the brain, CCL2 production is rapidly induced by a range of diverse inflammatory conditions, and is associated with the infiltration of blood-derived macrophages and activation of microglia [3,4]. Transgenic mice over-expressing *Ccl2 *in the central nervous system (CNS) exhibit a robust accumulation of macrophages in the brain [5,6], whereas mice deficient in the *Ccl2 *gene show reduced leukocyte infiltration after TBI, spinal cord injury and stroke [7-9]. Detection of elevated CCL2 in the serum and cerebrospinal fluid of severe TBI patients [9,10] corroborates a central role for this chemokine in post-traumatic neuroinflammation.

We recently identified an altered profile of cortical cytokine production in *Ccl2*-deficient mice subjected to a closed head injury model of focal TBI. Peak levels of interleukin (IL)-1α, IL-1β, IL-6, granulocyte-colony stimulating factor (G-CSF), IL-12(p40), CCL3 and CXCL1 were delayed and significantly exacerbated in *Ccl2*-/- mice compared to wild-type mice acutely post-injury, whilst the production of other inflammatory mediators including CCL5, interferon-gamma (IFNγ) and IL-2 had reduced production in chemokine-deficient animals [9]. These findings appear paradoxical to the delayed neuroprotection shown in *Ccl2*-/- mice, which had reduced macrophage accumulation and tissue damage associated with improved functional recovery over 4 weeks post-injury. We hypothesise that the altered cytokine network in the brains of *Ccl2*-/- mice after injury may indicate a previously unrecognised role for CCL2 as a modulator of acute CNS inflammation. Based on astrocytes being the main source of chemokines including CCL2 [11-14], it is conceivable that CCL2 may exert immunomodulatory effects on this cell type.

In the current study, we aimed to elucidate whether CCL2 regulates immune processes by investigating cytokine production from *Ccl2*-/- astrocytes compared to wild-type cells in response to inflammatory stimuli *in vitro*. We demonstrate that primary *Ccl2*-/- astrocyte cultures secrete exacerbated levels of chemokines and cytokines such as interleukin (IL)-6 compared to wild-type cells in response to lipopolysaccharide (LPS) and IL-1β stimulation. Furthermore, production of IL-6 induced by IL-1β in wild-type astrocytes was suppressed by prior incubation with exogenous CCL2. These results indicate a likely immunomodulatory role for CCL2 in astrocytic cytokine production. In combination with other recently identified functions of this chemokine in neurotransmission [15,16] and neuronal cell survival [17-19], these findings indicate that future application of therapeutics targeting CCL2-mediated leukocyte infiltration following TBI may have tangential effects in the injured brain.

## Methods

### Animals and reagents

The experimental procedure was approved by the Alfred Medical Research and Education Precinct (AMREP) Animal Centre, Melbourne, Australia. *Ccl2*-/- mice (B6.129S4-Ccl2tm1Rol/J) on a C57Bl/6 background were obtained from Jackson Laboratory (Maine, USA) and a breeding colony established at AMREP. C57Bl/6 mice were used as wild-type controls. Unless otherwise stated, all reagents were obtained from Invitrogen Laboratories, Carlsbad, CA or Sigma Aldrich, St Louis, MO.

### Isolation of primary mouse astrocytes

Primary astrocyte cultures were obtained from newborn (0-2 day old) mice as previously described [20,21]. Following isolation of cortices and removal of the meninges, dissociated cells were suspended in Dulbecco's Modified Eagle's Media (DMEM) containing 10% fetal bovine serum (FBS) and 0.25% gentamycin, and plated on 75 cm^2 ^flasks pre-coated with 0.1 mg/ml poly-L-lysine. Cultures were maintained for 7 days to generate a confluent glial culture.

Prior to trypsinisation, contaminating microglial cells were separated by vigorous mechanical agitation and removed by subsequent washing in Hank's Balanced Salt Solution (HBSS). Astrocytic monolayers were then dislodged from flasks by trypsinisation (0.25% trypsin in HBSS and 1 mM EDTA). Cells were seeded in either 24-well plates (1 × 10^5 ^cells/well) or 75 cm^2 ^flasks (5 × 10^5 ^cells/flask) and grown for 7-9 days until confluent prior to stimulation. Culture purity was determined by double-labelling immunohistochemistry for GFAP (1:1000; DAKO, Glostrup, Denmark) and CD11b (1:150; BD Pharminogen, San Diego, CA) to identify astrocytes (>95%) and microglia (<5%), respectively. The lack of *Ccl2 *mRNA amplified by quantitative PCR confirmed the absence of *Ccl2 *in gene-deficient cultures.

### Astrocyte stimulation

16 hours prior to stimulation, cultures were washed in HBSS and cultured with low-serum medium (DMEM containing 1% FBS). Astrocytes were then stimulated with either LPS (from *Escherichia coli *0111:B4 γ-irradiated; 0.1-100 μg/ml), mouse recombinant (r) IL-1β (<1 EU/μg endotoxin; Peprotech, Rocky Hill, NJ; 0.1-50 ng/ml) or rCCL2 (R&D Systems, Minneapolis, MN; 50-500 ng/ml) in low-serum (1%) DMEM. LPS and IL-1β in particular have been shown to induce cytokine production by cultured astrocytes across a wide range of concentrations, from 1 ng/ml to 1 mg/ml LPS [21-30] and between 0.1 - 100 ng/ml IL-1β [31-35]. In some experiments, cultures were pre-treated with rCCL2 (250 ng/ml) for 12 h, before the addition of LPS or IL-1β for a further 8 h. This experimental design was based on one previously used to demonstrate the regulation of macrophage cytokine production by the chemokine fractalkine [36]. Three separate cultures were stimulated, such that data represents the mean of three independent experiments. Parallel astrocyte cultures were incubated in medium alone as unstimulated controls. After stimulation (2 - 48 h) supernatant was collected, centrifuged (1000 rpm, 10 min at 4°C) and stored at -20°C until analysis, whilst cells were washed in HBSS and scrapped for collection, then centrifuged prior to storage of cell pellets at -80°C.

### Measurement of cytokine production in culture supernatants

Commercially-available mouse multiplex kits (LINCOplex; Millipore, Billerica, MA) were used to quantify the production of the cytokines IL-6 and TNF, as well as the chemokines CXCL1, CCL3 and CCL5, in culture supernatants following stimulation with LPS. These five inflammatory mediators are all reportedly produced by mouse, rat and human astrocytes, in response to a variety of stimuli including mechanical injury and pro-inflammatory cytokines. Mouse IL-6 secreted in response to IL-1β stimulation was measured using Quantikine Immunoassays (R&D Systems; lower detection limit <1.6 pg/ml).

### Quantification of gene expression by real-time reverse transcriptase polymerase chain reaction (qPCR)

RNA was extracted and isolated from astrocyte cells using the Purelink RNA Mini Kit (Invitrogen), including on-column DNase treatment, as per the manufacturers' instructions. RNA concentration and purity was determined using a Nanodrop1000 spectrophotometer (Thermo Fisher Scientific), with an absorbance ratio at 260 and 280 nm of >1.9 considered as highly pure RNA. One microgram of each sample was reverse transcribed into complementary-strand DNA (cDNA) using SuperScript™ III reverse transcriptase and oligo d(T)_20 _as the primer according to the manufacturers' protocol (Invitrogen). Quantitative PCR (qPCR) was performed using the TaqMan Universal master mix (Applied Biosystems, Foster City, CA) and TaqMan^® ^gene expression assay for mouse IL-6 (Mm01210733_m1, Applied Biosystems). Samples were assayed in triplicate on a 384 well plate using the 7900 Fast Real-Time PCR system (Applied Biosystems).

The widely-accepted comparative Ct (threshold cycle) method was used to perform relative quantification of qPCR results [37]. An arithmetic formula (fold difference = 2^-ΔΔCt^) was used to calculate the relative mRNA expression of stimulated cultures compared to unstimulated controls, after normalisation to levels of the housekeeping control gene, glyceraldehyde-3-phosphate dehydrogenase (GAPDH; Mm99999915_g1). Data are thus expressed as fold change compared to unstimulated wild-type cells.

### Statistical analysis

Statistical analysis was performed using Sigma Stat 2.03 software (SPSS Inc., Chicago, IL). Data shown represent means ± standard error of the mean (SEM) from at least three independent experiments. Two-way Analysis of Variance (ANOVA) tests were used to evaluate cytokine production across time or different stimulus concentrations, with overall significance for factors of strain and time/concentration reported as such. Tukey's test was employed for *post-hoc *comparisons between and within individual factors (indicated on figures by * and #), whilst differences between two factors (e.g. gene expression in LPS-stimulated wild-type and *Ccl2*-/- cells) were analysed by t-tests. When necessary, non-parametric data was normalised by transformation by natural log (ln) prior to statistical analysis. Statistical significance was considered at the 5% level (p < 0.05).

## Results

### Cytokine production was exacerbated in LPS-stimulated *Ccl2*-/- astrocytes

**Figure 1:**
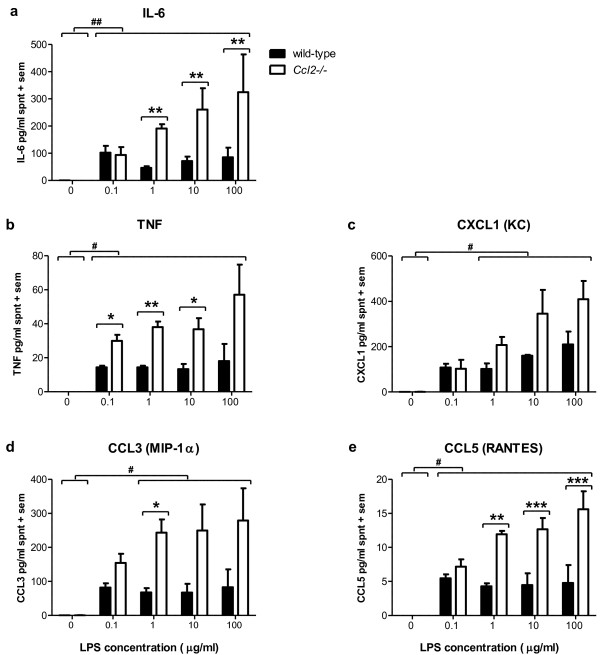
**Exacerbated cytokine production in *Ccl2*-/- astrocyte supernatant in response to LPS**. Levels of inflammatory mediators were measured by multiplex assay in astrocyte supernatants following incubation for 8 h with LPS (0-100 μg/ml). Production of IL-6 and TNF (a and b), as well as the chemokines CXCL1, CCL3 and CCL5 (c, d and e), was increased in response to LPS stimulation (p < 0.001, 2-way ANOVA effect of concentration), and was exacerbated in *Ccl2*-/- astrocyte cultures compared to wild-type (p < 0.01; 2-way ANOVA effect of strain). The hash symbol (#) represents post-hoc analysis of differences between cytokine/chemokine levels at increasing LPS concentrations (# p < 0.05; ## p < 0.01), whilst stars (*) annotate post-hoc analysis of differences between strains (* p < 0.05; ** p < 0.01; *** p < 0.001). Data represent mean + sem of three separate experiments assayed in duplicate. Spnt = supernatant.

Concentration of several cytokines and chemokines were measured in supernatant collected from wild-type and *Ccl2*-/- astrocyte cultures, to determine the effect of *Ccl2 *gene deletion on the inflammatory response to LPS stimulation. From preliminary experiments examining a time-course of LPS stimulation (range of 1 - 48 h), we determined that sub-maximal cytokine production was evident by 8 h (data not shown). Thus the concentration of IL-6, TNF, CXCL1 (KC), CCL3 (MIP-1α) and CCL5 (RANTES) was measured after 8 h of LPS exposure. As expected, cytokine production was at low or undetectable levels in unstimulated astrocyte cultures (0 μg/ml LPS) in both cell genotypes (figure 1).

In general, the cytokines IL-6 and TNF (figure 1a and 1b) were differentially stimulated in *Ccl2*-/- astrocytes across the range of LPS concentrations (0.1 - 100 μg), with exacerbated production compared to wild-type cells. For IL-6, no differences were noted between the strains at 0.1 μg/ml LPS, whereas from 1-100 μg/ml, whilst IL-6 production by wild-type cells reached a plateau, production by *Ccl2*-/- astrocytes increased dose-dependently (p < 0.01; 2-way ANOVA effect of strain). TNF secretion showed a similar pattern, with *Ccl2*-/- astrocytes showing significantly greater cytokine production from 0.1 μg/ml LPS onwards (p < 0.001; 2-way ANOVA effect of strain). In addition, production of the neutrophil chemoattractant CXCL1 (figure 1c) and two macrophage chemokines CCL3 and CCL5 (figure 1d and 1e) was significantly elevated in *Ccl2*-/- astrocytes (p < 0.01; 2-way ANOVA effect of strain), with the later being particularly exacerbated in gene-deficient cells at higher LPS concentrations of 10 and 100 μg/ml (p < 0.001; *post-hoc*). CCL5 was secreted at notably lower levels (~ 5-15 pg/ml) compared to CCL3 and CXCL1 (~ 100-400 pg/ml) in cell supernatant, however its concentration was consistently enhanced in *Ccl2*-/- compared to wild-type astrocytes ((p < 0.01; 2-way ANOVA effect of strain).

Importantly, whilst cytokine and chemokine production was induced in wild-type cells by LPS treatment, this effect appeared to plateau from 0.1 μg/ml LPS, after which increasing concentrations of the stimulus did not augment mediator secretion further. In contrast, *Ccl2*-/- astrocytes exhibited an increasing dose-dependent response, with the maximal LPS concentration used (100 μg/ml) inducing the greatest secretion of all five mediators (p < 0.001, 2-way ANOVA effect of concentration).

**Figure 2:**
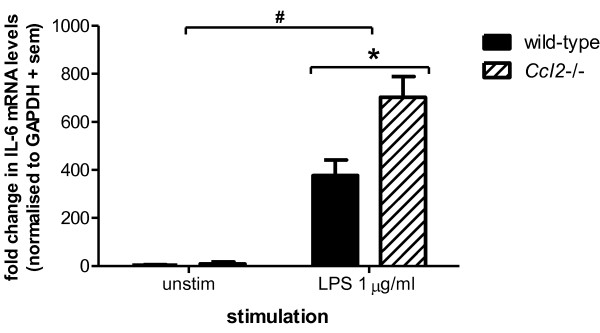
**IL-6 mRNA expression is exacerbated in *Ccl2*-/- astrocytes following LPS stimulation**. Expression of IL-6 mRNA was measured by qPCR in cell lysates following stimulation with 1 μg/ml LPS for 8 h. Whilst mRNA levels were minimal in unstimulated cells, IL-6 expression was strongly induced by LPS exposure in astrocytes from both strains (# p < 0.001; 2-way ANOVA effect of treatment). Furthermore, significantly higher expression of IL-6 mRNA was detected in *Ccl2*-/- astrocytes after LPS treatment compared to LPS-treated wild-type cells (* p < 0.05). Data represent mean + sem of three separate experiments assayed in triplicate.

IL-6 is a pleiotropic cytokine with diverse roles in the injured CNS [38]. Both in the injured brain and in stimulated astrocyte cultures, IL-6 is produced at high concentrations as compared to other mediators [9,22,26,31,39]. Furthermore, glial-derived IL-6 is pivotal in multiple neuroinflammatory cascades, as *Il-6*-gene deficient astrocytes *in vitro *show an altered cytokine profile compared to wild-type control cultures [23]. Therefore, subsequent experiments focused on IL-6 production to enable better detection of selective responses resulting from stimulation with LPS or IL-1β. The differential production of IL-6 by *Ccl2*-/- astrocytes was thus also examined at the transcriptional level, by quantifying gene expression in stimulated cells by qPCR (figure 2). Whilst minimal IL-6 was detectable in unstimulated cells from either strain, following incubation with LPS (1 μg/ml), IL-6 mRNA levels were increased 125-fold in wild-type astrocytes. Furthermore, IL-6 expression in *Ccl2*-/- astrocytes was significantly enhanced, with an additional upregulation of approximately 50% compared to wild-type cells (p < 0.05).

### IL-6 production by IL-1β-stimulated *Ccl2*-/- astrocytes is concentration-dependent

**Figure 3:**
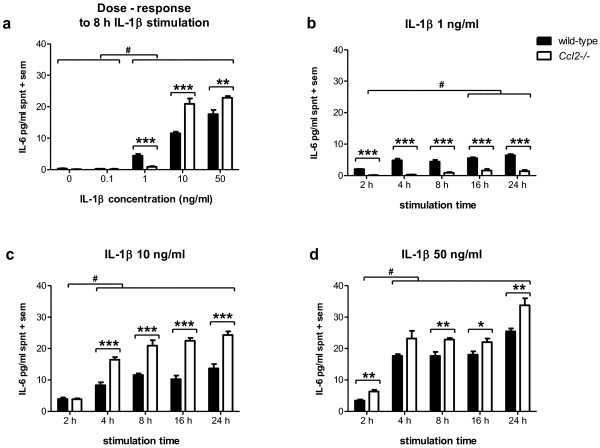
**IL-6 production by IL-1β-stimulated astrocytes is concentration-dependent and enhanced in *Ccl2*-/- cells**. IL-6 levels were measured in astrocyte supernatant following stimulation with IL-1β at various concentrations (a) and time periods (b-d). At 1 ng/ml IL-1β, wild-type cells produced significantly more IL-6 compared to *Ccl2*-/- astrocytes from 2-24 h exposure, albeit at low levels. In contrast, at 10 and 50 ng/ml IL-1β, *Ccl2*-/- astrocytes produced significantly higher levels of IL-6 across the time course. Stars annotate p-values representing differences between strains from post-hoc analysis (*p < 0.05; **p < 0.01; ***p < 0.001), whilst the hash symbol designates differences between stimulation times/concentrations (# p < 0.001). Data represent mean + sem of three separate experiments assayed in duplicate. Spnt = supernatant.

Astrocyte cultures were next treated with the pro-inflammatory cytokine IL-1β, to determine whether the differential response of *Ccl2*-/- cells to LPS could also be induced by a milder stimulus with close relevance to post-traumatic neuroinflammation [40,41]. Measurement of IL-6 in the supernatant surprisingly revealed lower levels of IL-6 produced by *Ccl2*-/- astrocytes compared to wild-type cells after 8 h exposure to 1 ng/ml IL-1β (p < 0.001; *post-hoc*). Inversely, when using higher concentrations of IL-1β (10 or 50 ng/ml), a greater response was detected in *Ccl2*-/- astrocytes compared to that observed in wild-type cells (p < 0.001 and p < 0.01 respectively; *post-hoc*) (figure 3a).

To further investigate this incongruity, wild-type and *Ccl2*-/- astrocyte cultures were treated with IL-1β at 1, 10 or 50 ng/ml and compared across a time course of 2 - 24 h. IL-1β at 1 ng/ml simulated low levels of IL-6 by astrocytes from both strains (< 7 pg/ml in wild-type cultures and < 2 pg/ml in *Ccl2*-/- cultures), with IL-6 secretion being consistently higher (~2-8 pg/ml) in wild-type astrocytes compared to *Ccl2*-/- cells (<2 pg/ml; p < 0.001; 2-way ANOVA effect of strain), which was evident at all time points from 2 h onwards (p < 0.001; *post-hoc*) (figure 3b). In contrast, using a higher concentration of IL-1β (10 ng/ml) induced a greater production of IL-6 compared to 1 ng/ml stimulation in both strains. IL-6 secretion was increased in wild-type astrocytes at 4 h compared to 2 h, however, little additional secretion was seen with an increased length of exposure (4-24 h) (figure 3c). On the contrary, IL-6 induction in *Ccl2*-/- astrocytes was more robust across the time course, with production exacerbated above levels in wild-type astrocytes steadily increasing over time to a maximum after 24 h stimulation (p < 0.001; post-hoc).

Similarly, stimulation with IL-1β at 50 ng/ml induced a further enhanced response from *Ccl2*-/- astrocytes compared to wild-type (2-way ANOVA effect of strain). This was evident as early as 2 h (6.32 ± 0.57 pg/ml; p < 0.01, *post-hoc*) and maintained across the time course, with IL-6 secretion peaking in *Ccl2*-/- astrocytes at 24 h (33.74 ± 2.24 pg/ml) (figure 3d). Interestingly, differences between the strains were less pronounced at 50 ng/ml compared to IL-6 production induced by IL-1β at 10 ng/ml.

In summary, the enhanced production of IL-6 in *Ccl2*-/- astrocytes following IL-1β stimulation was dependent on the concentration of the stimulus, but independent of the length of exposure time.

### Exogenous CCL2 does not induce cytokine release in astrocytes

Given the differential production of cytokines in the absence of CCL2, we postulated that treatment with the exogenous recombinant chemokine may modulate cytokine secretion.

To ascertain this hypothesis, wild-type cultures were incubated with mouse rCCL2 for 12 h with a range of concentrations (50, 100, 250 and 500 ng/ml), and the production of IL-6, TNF, CXCL1, CCL3 and CCL5 was measured in the collected supernatant. Even at the highest concentration of rCCL2, production of TNF, CXCL1, CCL3 and CCL5 were below detectable levels (reported as 0.9, 1.2, 2.8 and 0.7 pg/ml respectively). IL-6 concentrations were barely detectable in unstimulated cultures, and increasing concentrations of rCCL2 had no additional effect on IL-6 secretion (p = 0.700; one-way ANOVA; data not shown). Similarly, IL-6 production from *Ccl2*-/- astrocytes following rCCL2 treatment was negligible (<0.2 pg/ml; data not shown).

### rCCL2 reduces IL-1β induction of IL-6 production and expression

**Figure 4:**
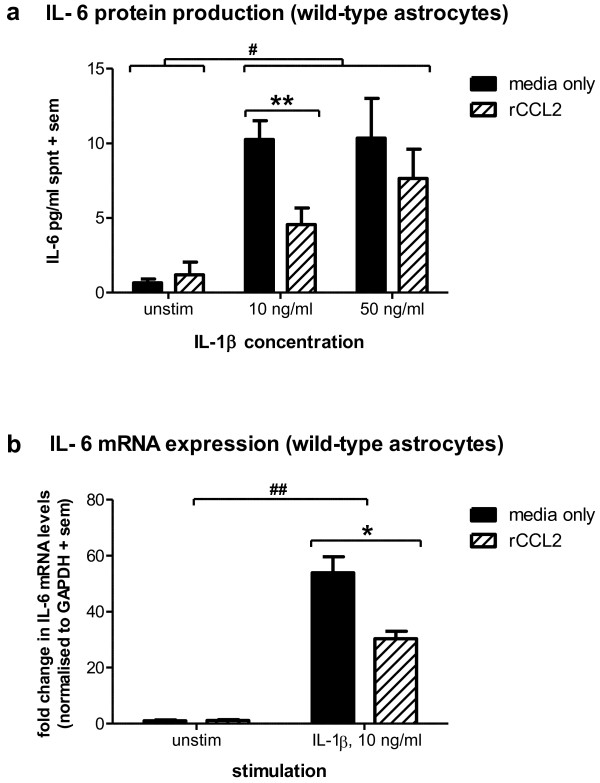
**Pre-treatment with rCCL2 inhibits IL-1β induction of IL-6**. Wild-type astrocytes were firstly pre-incubated with rCCL2 (250 ng/ml) or media only for 12 h before the addition of IL-1β (10 or 50 ng/ml) for a further 8 h. IL-6 protein secretion in supernatant was detected by ELISA (a) and mRNA levels were measured in cell lysates by qPCR (b). IL-6 protein production increased in response to IL-1β stimulation (# p < 0.01; 2-way ANOVA). Pre-treatment with rCCL2 effectively reduced IL-6 production induced by IL-1β at 10 ng/ml (** p < 0.01). IL-6 mRNA was similarly increased after incubation with IL-1β (## p < 0.001; 2-way ANOVA). This response was reduced by pre-treatment with rCCL2 (* p < 0.05). Data represent mean + sem of three separate experiments assayed in triplicate.

Since IL-1β stimulated a differential IL-6 production in wild-type and *Ccl2*-/- astrocytes, we hypothesised that pre-treatment with rCCL2 prior to exposure to IL-1β may attenuate this cytokine response. Therefore, cultures from wild-type mice were firstly incubated for 12 h with either media only or rCCL2 (250 ng/ml; 8 h), before the addition of IL-1β (10 or 50 ng/ml) as the stimulus. As expected, IL-6 measured in the supernatant of wild-type cultures was increased following stimulation with IL-1β at both 10 and 50 ng/ml (p < 0.01; 2-way ANOVA effect of concentration). Importantly, rCCL2 pre-treatment significantly reduced IL-6 production induced by IL-1β compared to pre-treatment with media only. This was most notably at 10 ng/ml, whereby ~ 50% less IL-6 was detected in supernatant from rCCL2-treated cultures (p < 0.01; *post-hoc*; figure 4a).

IL-6 gene expression was next measured by qPCR in wild-type cells collected post-stimulation (figure 4b). As for supernatant measurements, cultures were incubated for 12 h with either media only or rCCL2 (250 ng/ml), before the addition of IL-1β (10 ng/ml; 8 h). 10 ng/ml of IL-1β was chosen for investigation, as it was the concentration at which we observed the greatest effect of rCCL2 inhibition (figure 4a). Consistent with protein production, the levels of IL-6 mRNA were consistently low in unstimulated cells regardless of pre-incubation with either media alone or rCCL2. Also consistent with the protein data, IL-1β stimulation robustly increased IL-6 expression by ~ 50 fold compared to levels in unstimulated cells (p < 0.001; 2-way ANOVA). In contrast, rCCL2 pre-treatment significantly inhibited IL-6 mRNA levels in response to IL-1β by 44% compared to pre-treatment with media alone (p < 0.05), corresponding with the reduced IL-6 protein detected under the same conditions.

## Discussion

Investigations into inflammatory neuropathologies such as multiple sclerosis and stroke have demonstrated neuroprotection resulting either from interference with the *Ccl2 *gene, by administration of neutralising anti-CCL2 antibodies, or targeting the CCR2 receptor [7,42-44]. Besides the chemotactic ability of CCL2 to drive the migration of monocytic cells into the brain, recent evidence has shown that CCL2 also has alternative neuroactive properties in the CNS [17-19,45]. Furthermore, research by our group and others have reported alterations in the inflammatory cytokine response and tissue damage in *Ccl2*-deficient mice after TBI or stroke, prior to the onset of leukocyte infiltration [7,9]. The purpose of our current study was thus to investigate whether CCL2 can influence astrocyte-specific cytokine secretion following an immune challenge *in vitro*.

The production of the cytokine IL-6 and chemokines CXCL1 and CCL3 was significantly exacerbated in the supernatant of *Ccl2*-/- astrocytes following stimulation with LPS, corresponding to the delayed and enhanced secretion detected in the injured mouse brain [9]. TNF, whose production was elevated in the brains of *Ccl2*-/- mice across a time course of 2-24 h after experimental TBI, was also elevated in LPS-stimulated *Ccl2*-/- astrocytes *in vitro*. Differences in cytokine protein levels in wild-type and *Ccl2*-/- astrocyte cultures are likely due to variations in gene expression, as we also detected an enhanced elevation of IL-6 mRNA levels in *Ccl2*-/- astrocytes compared to wild-type cells.

Interestingly, the macrophage chemoattractant CCL5 was produced at lower levels in the brains of *Ccl2*-/- mice compared to wild-type after TBI, however, gene-deficient astrocytes had a profile of exacerbated CCL5 production. Particularly considering the low concentration of CCL5 detected in culture supernatant following LPS stimulation compared to the other cytokines measured, it is possible that this chemokine may be primarily produced during neuroinflammation by other CNS cells such as microglia, as has been previously suggested [35,46,47].

For subsequent experiments we focused specifically on astrocyte production of IL-6, as this multi-functional cytokine is central to CNS inflammation and degeneration following TBI [40,41], and is secreted *in vitro *in response to mechanical injury and exposure to inflammatory cytokines [22,26,29,31,48,49]. Consistent with the LPS-stimulated phenotype, a similarly enhanced response by *Ccl2*-/- astrocytes was evident following stimulation with the pro-inflammatory cytokine IL-1β at both 10 and 50 ng/ml. Interestingly, this characteristic exacerbated response was not evident in *Ccl2*-/- astrocytes at a lower concentration of IL-1β (1 ng/ml), when wild-type cells produced a higher level of IL-6. This finding may reflect what we observed *in vivo*, where secretion of IL-6 was similar in wild-type and *Ccl2*-/- mice early after injury (2-4 h) before a delayed and enhanced response in *Ccl2*-/- mice occurred at 12 h, corresponding to the time of maximal IL-1β in the lesioned cortex [9].

There have been several other studies suggesting that *Ccl2*-deficiency deregulates inflammatory cytokine synthesis in the CNS, primarily restricted to *in vivo *rodent models. Contrary to our findings, Hughes and colleagues (2006) reported that IL-6 and IL-1β were significantly reduced in the brains of *Ccl2*-/- mice after induction of ischaemic stroke [7]. Similarly, lower levels of IL-1β and TNF were induced in *Ccl2*-/- mice compared to wild-type after a striatal injection of LPS [50]. A third study also using LPS as the stimulus found that peripheral administration resulted in elevated IL-1β and TNF levels in serum of *Ccl2*-/- mice, whereas cytokine production in the brain was attenuated [51]. It is worth noting that all three of these studies considered only one time point (6 h) after stroke or LPS stimulation. Since we have shown here that alterations in cytokine production resulting from *Ccl2*-deficiency are time and concentration-dependent both *in vivo *and *in vitro*, it is possible that these studies do not comprehensively reflect the overall neuroinflammatory response specific to this strain. Importantly, our current work is the first to examine *Ccl2*-deficient anomalies in cytokine production or synthesis in depth by using an *in vitro *system of astrocyte activation.

Previous studies have suggested that the early and robust upregulation of CCL2 in neuropathologies such as TBI may indicate a central role for this chemokine in initiating the cytokine response [7]. Given that cytokine production in the brain coincides with CCL2 upregulation and most often precedes evidence of microglial activation or macrophage infiltration from the periphery, it is unlikely that the influence of CCL2 on acute cytokine production is dependent on the chemoattraction of leukocytes and subsequent secretion from these cells [51]. Furthermore, investigation of the macrophage and microglial marker F4/80 in the brains of *Ccl2*-/- mice early after injury or disease onset has failed to detect any difference from wild-type mice, suggesting that the contribution of CCL2 to acute neuropathology may be independent to its classic actions as a chemokine [7,50,52]. Given that incubation of cultured astrocytes with rCCL2 alone does not induce production of IL-6, TNF, CXCL1, CCL3 or CCL5 above levels in unstimulated cells, it is also unlikely that CCL2 directly stimulates cytokine production from astrocytes.

Alternatively, based on our findings of exacerbated cytokine production in the brains of injured *Ccl2*-deficient mice and immune-challenged *Ccl2*-deficient astrocytes, we hypothesised that CCL2 may act during early immunoactivation in an inhibitory manner. Interestingly, CCL2 has recently been proposed to protect neurons from cell death induced by N-methyl-D-aspartate, glutamate, β-amyloid and human immunodeficiency virus-mediated Tat toxicity [17-19]. Further prompting our own investigations into alternative properties of CCL2, Rankine and colleagues (2006) previously showed that co-administration of LPS and rCCL2 into the mouse brain did not have a synergistic pro-inflammatory effect as they had hypothesised - instead, the addition of rCCL2 in combination with LPS resulted in a non-significant trend towards reduced IL-6 production.

Also relevant to our findings, Zisman and colleagues showed that treatment with anti-CCL2 antibodies prior to an intraperitoneal LPS challenge increased plasma TNF and IL-12 levels, whilst administration of rCCL2 reduced pro-inflammatory cytokine production by 20-35% [53]. Similarly, in cultured astrocytes we have demonstrated that pre-exposure to rCCL2 was sufficient to reduce the IL-1β-stimulated production of IL-6 in culture supernatant. Furthermore, this inhibitory effect was also evident at a transcriptional level, as the increase in IL-6 mRNA expression induced by IL-1β stimulation was significantly lower in rCCL2-treated cultures. To our knowledge, this is the first *in vitro *evidence that CCL2 can act on astrocytes in an immunomodulatory manner to down-regulate cytokine expression and production.

One limitation of this study is the relative simplicity of the culture system used. It must be noted that cultured primary astrocytes may respond quite differently in isolation compared to *in vivo*, where they may be influenced by interactions with adjacent endothelial cells, microglia and extracellular matrix components. However, the consistency between our findings in cultured astrocytes and the mouse cortex *in vivo *[9] supports the use of primary astrocyte cultures as an appropriate model system.

The mechanisms underlying a novel role for CCL2 in CNS cytokine production need to be further elucidated. Whilst CCL2 mediates immune cell chemotaxis by binding to the CCR2 receptor, the presence of CCR2 on astrocytes in the brain remains controversial [14,54-57]. Quinones and colleagues recently found that CCL2 treatment *in vitro *can induce glial cell migration and inhibit the spontaneous apoptosis of mouse astrocytes in a dose-dependent manner. Whilst the latter pro-survival effect was shown to be dependent on signaling via CCR2, residual chemotaxis of *Ccr2*-/- astrocytes in response to CCL2 may indicate the presence of an alternative receptor for this chemokine on primary astrocytes [58]. Whether CCR2 is the primary receptor involved in CCL2-modulation of cytokine production, and which intracellular pathways are responsible for the reduction in cytokine expression observed in IL-1β-stimulated astrocytes pre-exposed to rCCL2, remain unanswered. The use of specific neutralising antibodies, or the recently generated cell-conditional CCL2 knockout mice in which gene deletion is specific to either astrocytes or endothelial cells, may prove essential in answering these questions and eliminating potential confound resulting from global gene deficiency [59].

Lastly, the consequences of an altered cytokine environment in the presence or absence of CCL2 remain unclear. Of note, *Ccl2*-/- mice show long-term improvements in neurological recovery and reduced tissue damage following experimental TBI, despite the aberrations in early cytokine secretion [9]. Given the acute nature of cytokine production in the injured brain, it is feasible that the aberrant, exacerbated cytokine profile seen in *Ccl2*-/- mice may contribute to early post-traumatic degeneration in this strain, which may compensate for the lack of CCL2 and contribute to early leukocyte infiltration. We hypothesise that CCL2 may have differential acute and long-term effects during neuropathology, as has been indicated for TNF [60,61]. A deeper understanding of the consequences of CCL2 deletion in CNS inflammation and cytokine production may allow us to better tailor therapeutic approaches to the appropriate time post-injury. Thus further studies are necessary to elucidate the biological and physiological relevance of these findings.

## Conclusions

In the current study we have employed an *in vitro *model of astrocyte activation to characterise the *Ccl2*-/- genotype-specific profile of cytokine and chemokine secretion as observed in the injured brains of *Ccl2-/- *mice. Our findings provide evidence that astrocytes are primarily responsible for exacerbated cytokine response in the absence of CCL2. Furthermore, we present data showing that rCCL2 can inhibit the astrocytic production of inflammatory cytokines in response to IL-1β stimulation. In light of these findings, we propose that CCL2 plays a novel, immunomodulatory role in the inflamed CNS by altering cytokine production in astrocytes, and that this effect is relative to the concentration of the stimulus present. This study highlights the complexity of chemokine - cytokine interactions in the CNS, and suggests that CCL2 may affect multiple cytokine pathways which could participate in neurodegenerative processes. Our data indicate that chemoattraction of immune cells is only one of the functions performed by CCL2 in the injured CNS, and that further investigation is required before targeting of this chemokine as a means of neuroprotection for neuropathologies in which CCL2 upregulation is a characteristic feature.

## Completing interests

The authors declare that they have no competing interests.

## Authors' contributions

BDS participated in the project design, performed the astrocyte culture experiments including astrocyte isolation, immunoassays, qPCR, data acquisition and analysis, and drafted and revised the manuscript. TF assisted with the design, coordination and interpretation of qPCR experiments and manuscript revisions. CMK conceived the study, assisted with its design and data analysis, and helped draft the manuscript. All authors read and approved the final manuscript.
